# *Plasmodium* transmission blocking activities of *Vernonia amygdalina* extracts and isolated compounds

**DOI:** 10.1186/s12936-015-0812-2

**Published:** 2015-07-25

**Authors:** Solomon M Abay, Leonardo Lucantoni, Nisha Dahiya, Geme Dori, Edson G Dembo, Fulvio Esposito, Guilio Lupidi, Sonny Ogboi, Robert K Ouédraogo, Annamaria Sinisi, Orazio Taglialatela-Scafati, R Serge Yerbanga, Massimo Bramucci, Luana Quassinti, Jean Bosco Ouédraogo, George Christophides, Annette Habluetzel

**Affiliations:** School of Pharmacy, University of Camerino, Piazza dei Costanti, 62032 Camerino, MC Italy; School of Medicine, College of Health Sciences, Addis Ababa University, Addis Ababa, Ethiopia; Institut de Recherche enSciences de la Santé, Direction Régionale de l’Ouest, Bobo-Dioulasso, Burkina Faso; Department of Pharmacy, University of Naples Federico II, Via Montesano 49, 80131 Naples, Italy; Department of Life Sciences, Imperial College London, London, UK; Discovery Biology, Eskitis Institute for Drug Discovery, Griffith University, Nathan, QLD 4111 Australia

**Keywords:** Malaria transmission blocking, Gametocytes, Sporogonic stages, *Plasmodium*, Phytomedicine, *Vernonia amygdalina*, Sesquiterpene lactone

## Abstract

**Background:**

Medicinal plants are a validated source for discovery of new leads and standardized herbal medicines. The aim of this study was to assess the activity of *Vernoniaamygdalina* leaf extracts and isolated compounds against gametocytes and sporogonic stages of *Plasmodiumberghei* and to validate the findings on field isolates of *Plasmodium falciparum*.

**Methods:**

Aqueous (Ver-H_2_O) and ethanolic (Ver-EtOH) leaf extracts were tested in vivo for activity against sexual and asexual blood stage *P. berghei* parasites. In vivo transmission blocking effects of Ver-EtOH and Ver-H_2_O were estimated by assessing *P. berghei* oocyst prevalence and density in *Anopheles stephensi* mosquitoes. Activity targeting early sporogonic stages (ESS), namely gametes, zygotes and ookinetes was assessed in vitro using *P. berghei* CTRP_p_.GFP strain. Bioassay guided fractionation was performed to characterize *V.amygdalina* fractions and molecules for anti-ESS activity. Fractions active against ESS of the murine parasite were tested for ex vivo transmission blocking activity on *P.falciparum* field isolates. Cytotoxic effects of extracts and isolated compounds vernolide and vernodalol were evaluated on the human cell lines HCT116 and EA.hy926.

**Results:**

Ver-H_2_O reduced the *P. berghei* macrogametocyte density in mice by about 50% and Ver-EtOH reduced *P. berghei* oocyst prevalence and density by 27 and 90%, respectively, in *An.stephensi* mosquitoes. Ver-EtOH inhibited almost completely (>90%) ESS development in vitro at 50 μg/mL. At this concentration, four fractions obtained from the ethylacetate phase of the methanol extract displayed inhibitory activity >90% against ESS. Three tested fractions were also found active against field isolates of the human parasite *P. falciparum*, reducing oocyst prevalence in *Anopheles coluzzii* mosquitoes to one-half and oocyst density to one-fourth of controls. The molecules and fractions displayed considerable cytotoxicity on the two tested cell-lines.

**Conclusions:**

*Vernonia amygdalina* leaves contain molecules affecting multiple stages of *Plasmodium*, evidencing its potential for drug discovery. Chemical modification of the identified hit molecules, in particular vernodalol, could generate a library of druggable sesquiterpene lactones. The development of a multistage phytomedicine designed as preventive treatment to complement existing malaria control tools appears a challenging but feasible goal.

**Electronic supplementary material:**

The online version of this article (doi:10.1186/s12936-015-0812-2) contains supplementary material, which is available to authorized users.

## Background

Despite intensive efforts to control malaria, the disease continues to be one of the greatest health problems faced by sub-Saharan African countries [1]. About 207 million clinical cases of malaria occurred in 2012 according to WHO estimates, maintaining its deplorable ranking as one of the top killer diseases [2]. Among the drugs currently used for malaria case management, artemisinin based combination therapy (ACT) [3] and primaquine [4] have impact on malaria transmission, by reducing the infectiousness of individuals to mosquitoes [5]. Clinical evidence indicate that activity of artemisinin derivatives is restricted to developing gametocytes, whereas primaquine is able to hit also the circulating mature gametocytes [6]. On the basis of the drugs’ respective activity profiles, the World Health Organization (WHO) recommends the addition of a single dose of primaquine to the ACT course for the management of uncomplicated *Plasmodiumfalciparum* malaria in areas threatened by artemisinin resistance and/or in pre-elimination phase [7]. However, a viable alternative to primaquine is urgently needed, considering that this compound can provoke haemolytic anaemia in patients with glucose-6-phosphate dehydrogenase deficiency [8]. Among the various available approaches, the exploration of empirically effective and chemically characterized anti-malarial plants represents a valid strategy for the discovery of new druggable compounds active against gametocytes and/or sporogonic stages for the development of multi-stage combination medicines.

The connection between medicinal plants and successful anti-malarial drug discovery dates back to 1820, time of quinine isolation from *Cinchona* bark [9] and continues to the current time, as witnessed by the development of various potent forms of ACT based on semisynthetic derivatives of artemisinin, a highly oxygenated sesquiterpene isolated as active principle of *Artemisia annua* [10]. The multistage activity of artemisinin derivatives on asexual blood stages and early gametocytes [11], has encouraged researchers to explore molecules of plant origin seeking not only activity on parasite stages developing in the vertebrate host, but also transmission blocking effects against the sporogonic stages developing in the mosquito vector. Recently, Lucantoni et al. demonstrated the in vivo transmission blocking property of azadirachtin A enriched formulation (NeemAzal^®^) on *Plasmodium berghei* sporogonic development in *Anopheles stephensi* mosquitoes [12]. When administered to mice at an azadirachtin A dose of 50 mg/kg, the commercial *Azadirachta indica* seed extract [13] was found to completely block parasite development in the vector. Azadirachtin A has been shown to inhibit the formation of flagellate microgametes from microgametocytes with an IC_50_ of 3.5 µM [14], suggesting that microgametogenesis is a main target process of NeemAzal^®^ transmission blocking action. The transmission blocking activity of this azadirachtin A rich neem product was recently confirmed in the human parasite *P.falciparum* by studies conducted in Burkina Faso on field isolates: gametocytaemic blood supplemented with 70 ppm NeemAzal^®^ and membrane fed to *Anopheles coluzzii* mosquitoes completely inhibited oocyst development and therefore infection in mosquitoes [15].

In many African countries, where malaria is endemic and in particular in rural areas where access to modern health care facilities is often hindered, traditional practices still play an important role. People give preference to the use of herbal remedies for several reasons including: easier access, lower cost, lack of awareness about modern drugs and belief that the use of traditional medicine is more safe and effective [16]. Currently, standardized anti-malarial phytomedicines are officially commercialized in various malaria endemic countries over the world, namely China, Ghana, India, Mali and Burkina Faso. Among few such products for which clinical research has been conducted, encouraging results were obtained with Qing hao (*Artemisia annua*, Democratic Republic of Congo trials), Totaquina (*Cinchona* spp., Multicounty trials) and Phyto-laria (*Cryptolepis sanguinolenta*, Ghana trial) showing a parasite clearance at days 5–7 after treatment of 70–100, 92–100 and 100%, respectively. These findings support their use as complementary tools to the conventional anti-malarial interventions or as alternative treatments in the absence of anti-malarial drugs [17].

In sub-Saharan countries, including Ethiopia, leaves of *Vernonia amygdalina* (Asteraceae) are used for the treatment and prevention of malaria [18–21]. The anti-plasmodial activity of this small shrub has been confirmed by several in vitro [22–26] and in vivo studies [27–30]. In a 4-day suppression test involving the administration of extracts immediately after mouse infection with *P. berghei*, a parasitaemia suppression of 67% was demonstrated for ethanolic and methanolic extracts at doses of 500 mg/kg (s.c.) and 1,000 mg/kg (p.o.), respectively [27, 31], while an aqueous extract given to mice at 125 mg/kg (p.o.) caused a 63% reduction of parasitaemia [28]. Employing the Rane test that involves the administration of extracts 3 days post-infection for the evaluation of curative efficacy, 200 mg/kg (i.p.) of aqueous [30] and 500 mg/kg (s.c.) of ethanolic extract [27] suppressed parasitaemia by 74 and 71%, respectively. Interestingly, in a clinical trial examining the efficacy of an infusion of fresh *V. amygdalina* leaves in patients with uncomplicated falciparum malaria, an adequate clinical response was reported in 67% of the cases. However, complete parasite clearance occurred in only 32% of patients with adequate clinical response, and among these, recrudescence occurred in 71% [31].

Several secondary metabolites have been isolated and characterized from *V.amygdalina* leaves, such as sesquiterpene lactones [32–35], steroidal saponins (vernoniosides) [36], and flavonoids (luteolin and its glycosides) [37]. The class of sesquiterpene lactones is probably the most peculiar of this plant and it includes the highly oxygenated derivatives vernolide [32], vernodalol [32], vernodalinol [33], epivernodalol [34], vernodalin and vernomygdalin [35] and vernolepin [38]. Vernolide and vernodalin exhibited IC_50_ of 1.87 and 0.52 µg/mL, respectively, against *P.falciparum* blood stages in vitro [39]. In another study, IC_50_ values of 8.4, 4.0, 4.2 and 11.4 µg/mL were obtained for vernolide, vernodalin, vernodalol and hydroxyvernolide, respectively [40].

On the basis of the above-illustrated broad knowledge available on *V. amygdalina* as an anti-malarial plant—from its evidenced appreciable clinical efficacy to the partially characterized activity profile of some secondary metabolite—this plant has been selected as a valid candidate for investigation on potential inhibitors of *Plasmodium* transmission stages.

The present study was aimed at characterizing the plant extracts for stage specific effects on gametocytes and sporogonic stages and at identifying the responsible compounds through a bio-guided fractionation approach, then validating them on *P. falciparum* field isolates.

## Methods

### Plant material

#### Source

*Vernonia amygdalina* (Asteraceae) leaves were collected in the area of Karat town, located in Konso district, i.e., 600 km south of Addis Ababa, Ethiopia, after the rainy season (October 2011). A leaf specimen was used for taxonomic identification, and authenticated by Mr. Melaku Wondafrash, a plant taxonomist. A voucher specimen was deposited (Solomon-0l) at the National Herbarium, Addis Ababa University, Ethiopia.

#### Preparation of extracts and fractions, and isolation of compounds

*Vernoniaamygdalina* leaves were air-dried at room temperature in the shade and ground using a blender. Ground material (50 g) was macerated in ethanol (EtOH) for 24 h and filtered with filter paper (Whatman^®^ no. 1). Ethanol was removed using a rotary evaporator at 40°C under reduced pressure. The extract was further concentrated by freeze drying and stored at −20°C until use. To prepare the aqueous extract (Ver-H_2_O), 50 g of ground *V.amygdalina* leaves were macerated in distilled water for 24 h, then filtered and freeze dried. The percent yields of the aqueous and ethanol extractions were 14 and 8%, respectively.

To identify secondary metabolites responsible for transmission blocking activity, bio-guided fractionation was employed on a methanol (MeOH) extract of *V.amygdalina* leaves. Briefly, 450 g of leaf powder was macerated in 1 L of MeOH for 24 h for three times. The extract was then filtered and concentrated with rotary evaporator to obtain a dried MeOH extract (34.5 g, yield 7.6%). A portion of this extract (27.9 g) was then partitioned, first between water and ethyl acetate (EtOAc), and then between water and butanol (BuOH), to get the following phases: EtOAc (14.3 g), BuOH (4.9 g) and H_2_O (7.9 g). The EtOAc phase was selected for bioassay-guided fractionation. It was subjected to medium pressure liquid chromatography (MPLC) over a column packed with silica gel (230–400 mesh) and eluted with the following solvent gradient of increasing polarity: *n*-hexane, to *n*-hexane:EtOAc (9:1), *n*-hexane:EtOAc (4:1), *n*-hexane:EtOAc (7:3), *n*-hexane:EtOAc (3:2), *n*-hexane:EtOAc (1:1), *n*-hexane:EtOAc (7:11), *n*-hexane:EtOAc (1:4), EtOAc, EtOAc:MeOH (1:1) and finally MeOH. A total of 40 eluates of 250 mL each were collected and combined on the basis of thin layer chromatography behaviour, to get 14 fractions which were then tested for anti-plasmodial activities in vitro.

**Fig. 1:**
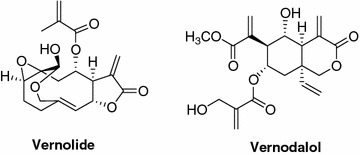
Chemical structure of vernolide and vernodalol.

The active fractions (fr. 11–14) were further purified by high performance liquid chromatography (HPLC) on a Knauer apparatus equipped with a refractive index detector and LUNA (5 µ, 250 × 4 mm Phenomenex) SI60 or Kinetex (2.6 µ, 100 × 4.60 mm Phenomenex) C18 columns. Fractions 11 and 12 were separated by HPLC (eluent *n*-hexane/EtOAc 75:25, flow 0.8 mL/min) to get pure vernolide (125.3 mg). Fractions 13 and 14 were separated by HPLC (eluent *n*-hexane/EtOAc 1:1, flow 0.8 mL/min) to obtain pure vernodalol (108.5 mg). The chemical structures of vernolide and vernodalol are reported in Fig. 1. These compounds were identified by comparison of their spectral data, particularly NMR (^1^H NMR at 500 MHz and ^13^C NMR at 125 MHz measured on Varian INOVA spectrometers) with those published in the literature [41, 42].

### Plasmodium species

The following three strains of the murine malaria parasite *P. berghei* were employed in transmission blocking experiments: (1) *P.berghei* ANKA strain (chloroquine and pyrimethamine-sensitive) [43, 44] for the assessment of extracts’ activity against sexual and asexual blood stages in vivo; (2) *P. berghei* GFPcon (chloroquine-sensitive and pyrimethamine-resistant) [43, 44] expressing a green fluorescent protein (GFP) at all life cycle stages, for the evaluation of extracts’ transmission blocking activity in vivo; (3) *P. berghei* CTRP_p_.GFP strain (chloroquine-sensitive and pyrimethamine-resistant), expressing GFP in zygotes and ookinetes [45, 46], for the assessment of extracts/fractions/molecules’ activity against the development of early sporogonic stages (ESS) in vitro. The *P.berghei* CTRP_P_.GFP strain was kindly provided by Prof. R.E. Sinden from Imperial College of London, UK.

*Plasmodiumfalciparum* parasites isolated from individuals living in a hyperendemic area of Burkina Faso were employed to confirm transmission blocking effects on the human parasite species. The details of ethical approval are presented below in the ethical clearance section.

### Mosquitoes

For the in vivo transmission blocking studies with *P. berghei*, *An. stephensi* mosquitoes were used as experimental vectors. The mosquito colony was maintained at a temperature of 30°C (±2°C), 12 h light/12 h dark cycle and 75–85% relative humidity in the insectary of the University of Camerino, Italy. Experiments were conducted with four to 5 days-old female mosquitoes that had been transferred to a 19°C chamber (temperature required for the development of *P.berghei* in the vector) 24 h prior to administration of infectious blood-meals.

For the ex vivo transmission blocking assay performed with *P.falciparum* field isolates, 4–5 days old *An. coluzzii* mosquitoes (previously classified as *Anopheles gambiae* form “M”) [47] were used. The colony, routinely reared in the insectary of the “Institut de Recherche en Sciences de la Santé”, Bobo-Dioulasso, Burkina Faso has been originally established from field collected mosquitoes in 2008.

### Mice

Eight to ten weeks old BALB/c mice, weighing between 18 and 25 g were used in this study. The mice were reared in the animal house of the University of Camerino at 24°C, 14 h light/10 h dark cycle and 70% relative humidity, fed on standard laboratory mice pellets (Mucedola s.r.l., Milano, Italy) and provided with tap water ad-libitum. Experimental animal rearing and handling were in compliance with the Italian Legislative Decree on the “use and protection of laboratory animals” (D. Lgs. 116 of 10/27/92) and in full adherence with the European Directive 2010/63/UE. The mice were narcotized with a 1:1 mixture of xylazine and acepromazine at 13% inoculate in 100 μL phosphate buffered saline (PBS) i.p. before any mosquito bites procedures. At the end of experiments, carbon dioxide inhalation was the method of humane euthanasia used for mice.

### Murine malaria model

The rodent in vivo malaria model consisting of *P. berghei* ANKA strain parasites, BALB/c mice as vertebrate hosts and *An. stephensi* mosquitoes as vectors, is routinely employed in drug discovery research for the assessment of in vivo transmission blocking and parasite suppression efficacy [12, 48–50] and has been validated by Coleman and colleagues [51]. This model was employed here to evaluate the in vivo transmission blocking and gametocytocidal activity of *V.amygdalina* extracts.

### Experimental procedures

#### Assessment of impact on parasite development in the vertebrate host and determination of gametocytocidal activity

Ethanolic (Ver-EtOH) and aqueous (Ver-H_2_O) extracts of *V.amygdalina* leaves at a dose of 500 mg/kg of mouse body weight were orally administered to experimental animals allocated to the treatment groups. This dosage corresponds approximately to the amount of the plant material taken up by malaria patients, according to the traditional medicine recipe [18]. For administration, Ver-EtOH was dissolved in distilled water containing 7.5% Tween 80 and 10% ethanol, while Ver-H_2_O was dissolved in distilled water only. Animals in the control group received a similar volume of the solvent used to dissolve the extracts. The daily dose of each extract was divided in two equal parts, and gavages were performed twice a day at an interval of 12 h, in a of 200 µL/mouse each. In each treatment and control group, six mice were used. Treatments were administered for a total of 9 days, starting 2 days before infection of the experimental mice through the bites of 12–14 *Anopheles* females harbouring sporozoites in their salivary glands [52]. On day 7 post-infection, thin blood smears were prepared and stained with 7.5% Giemsa solution (Merck, Germany) for 50 min. Smears were examined under a microscope with oil immersion objective (100×) to assess parasitaemia (all stages) and gametocyte densities. *Plasmodiumberghei* infected red blood cells (RBCs) were counted out of 100 total RBCs on three microscopic fields to determine parasitaemia. For low parasitaemias (<1%), up to 4,000 total erythrocytes have been counted. To estimate gametocytaemia, microgametocytes and macrogametocytes were counted in at least 16 fields against 2,500 infected RBCs.

#### Assessment of transmission blocking activity in vivo

**Fig. 2:**
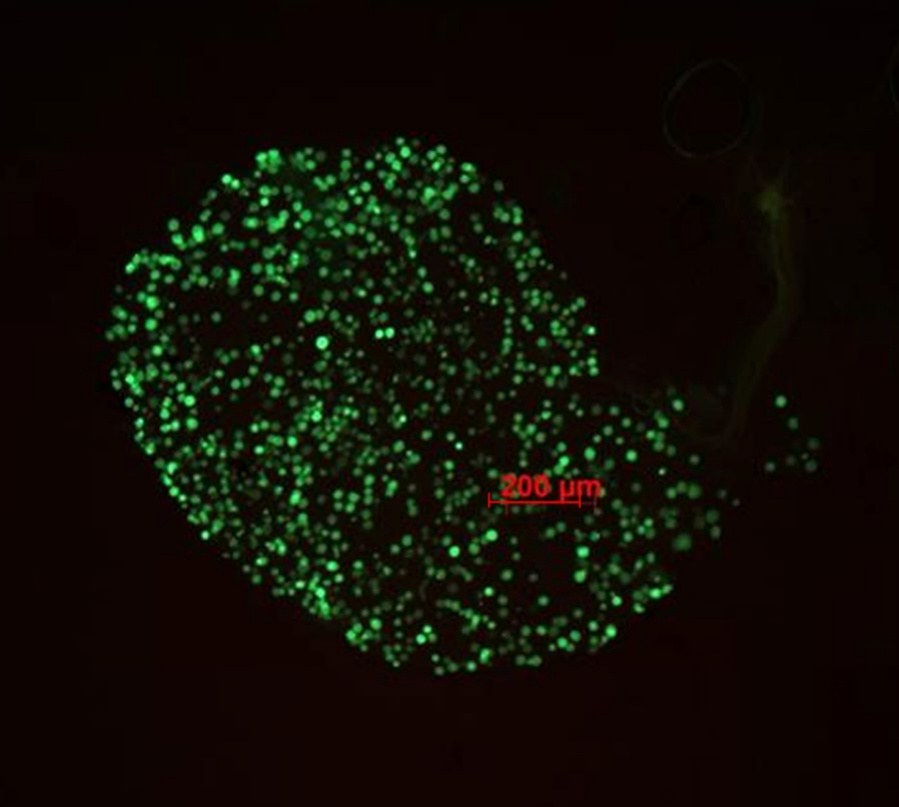
*Plasmodium berghei* GFPcon oocysts on mosquito mid-gut, 10 days after infection.

The in vivo transmission blocking effect of *V. amygdalina* was estimated by determining oocyst prevalence and density in mosquitoes fed on extract-treated gametocytaemic mice [12]. Briefly, mice were inoculated with 10^7^*P. berghei* GFPcon-infected red blood cells (RBCs) through the intraperitoneal (i.p.) route. On day 3 post-infection, animals allocated to the treatment groups were treated i.p. with 500 and 100 mg/kg (maximum tolerable dose) of Ver-H_2_O and Ver-EtOH, respectively, while control animals received 200 µL of the solvents used to dissolve the extracts. On day 4 post-infection, the treatment was repeated 1 h before the exposure of the mice to mosquitoes (day 4 gametocytes yield consistently high mosquito infections in this model). Mice were anaesthetized and kept on top of mosquito cages containing 150–200 *An.stephensi* females for 1 h. Unfed females were removed the following day. On day 10/11 after the blood meal, 17–34 mosquitoes from each cage were dissected. Oocyst prevalence and density were assessed by counting the number of green fluorescent oocysts on mosquito mid-guts under a fluorescence microscope (ZEISS Axio Observer Z1, FITC filter 9) (Fig. 2). Oocyst density was determined by calculating the geometric mean of the number of oocysts recorded on oocyst positive mosquitoes [53].

$$\begin{aligned} {\text{Geometric\,\,mean}} = antilog\left( {\left[ {\sum\nolimits_{i = 1}^{n} {logx} } \right]/n} \right),{\text{where}}\;{\text{n}} &= {\text{Number}}\;{\text{of}}\;{\text{oocyst}}\;{\text{positive}}\;{\text{mosquitoes}}, \hfill \\ x &= {\text{Oocyst}}\;{\text{number}}\;{\text{per}}\;{\text{mosquito}} \hfill \\ \end{aligned}$$

The percent reduction of oocyst density in treatment groups was calculated as follows:

$${\text{Percent reduction of oocyst density }} = \left( {1 - \frac{{{\text{average\,\,oocyst density in treated group}}}}{{{\text{average\,\,oocyst density in control group}}}}} \right) \times 100$$

The experiment was conducted in triplicate, using for each treatment group three mice and for each mouse one cage of mosquitoes.

### Evaluation of effects on early sporogonic stages in vitro

The impact of *V.amygdalina* on the development of ESS was evaluated according to the method described by Delves et al. [54] with slight modifications.

#### Source of gametocytes

**Fig. 3:**
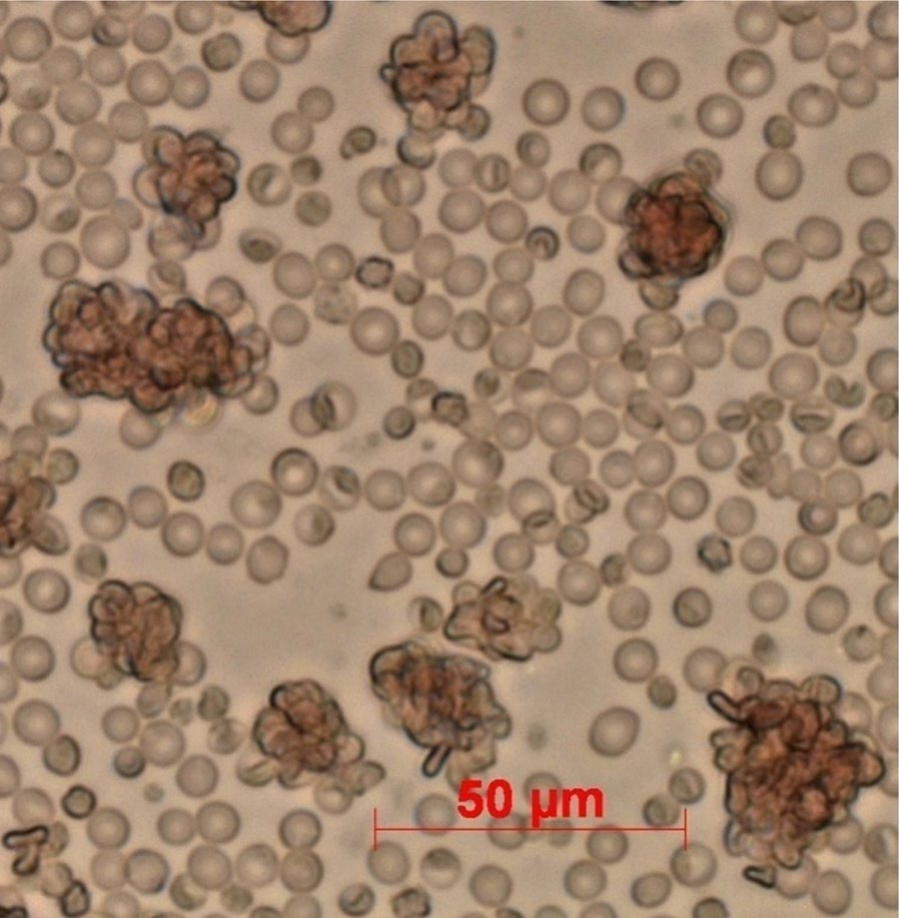
Exflagellation centres, vibrating accumulations of red blood cells attaching to microgametocytes with extruding motile, flagellate gametes. Photograph was taken after incubation of gametocytaemic blood in exflagellation medium for 20 min.

To stimulate erythropoiesis, mice were treated with phenylhydrazine (120 mg/kg i.p.) 4 days before being infected with *P. berghei* CTRP_p_.GFP through i.p. injection of 10^7^ infected RBCs. Gametocytaemia was checked 4 days post-infection by microscopic examination of thin blood films and the maturity of microgametocytes verified by testing their capacity to generate flagellate microgametes in an exflagellation assay. In brief, for each gametocytaemic mouse a drop of tail blood was diluted at a ratio of about 1:25 in exflagellation medium (RPMI 1640 containing 25 mM HEPES, 25 mM sodium bicarbonate, 50 mg/L hypoxanthine, 100 μM xanthurenic acid, pH 7.6–8). Then, 8 µL of the diluted blood sample were placed centrally on a hand-made chamber consisting of a microscope slide as a base, two cover slips placed on it laterally as spacers, and a third one put on the top to close the chamber. To avoid drying out of the 8 µL blood droplet, the chamber was sealed with a mixture of Vaseline and Tween 80 (approximately 1:2 ratio). After 20 min incubation at 19°C, slides were examined for exflagellation under the microscope (400× magnification) (Fig. 3). Mice with abundant exflagellation centres (more than 3 per 1,000 red blood cells) were selected for the ookinete development assay.

#### Ookinete development assay (ODA)

**Fig. 4:**
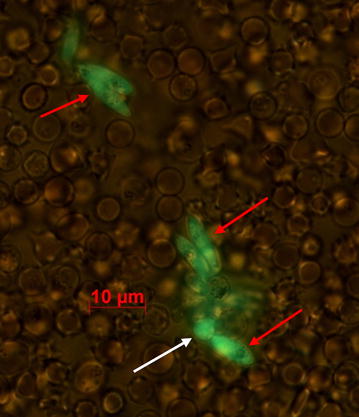
Early sporogonic stages: spherical zygote (*white arrow*) and elongated ookinetes (*red arrows*).

This assay allows the assessment of inhibition of the early sporogonic development in the vector, including male and female gametogenesis, zygote formation and ookinete maturation. To simulate the physicochemical conditions of the mosquito midgut environment in vitro, 180 µL of ookinete medium (exflagellation medium supplemented with 20% heat inactivated foetal bovine serum), 10 U/mL penicillin and 10 µg/mL streptomycin were added to a 96-well microplate (Nunc, Denmark). Dimethyl sulfoxide (DMSO, a solvent dissolve extracts/molecules) and PBS a solvent for the aqueous extract were used as controls, at a maximum concentration of 0.2%. Twenty microlitre of diluted *V.amygdalina* extracts, fractions or molecules were then added to the microplate wells to obtain the desired test concentrations (3–50 µg/mL). Then, 20 µL of blood obtained from gametocytaemic mice by cardiac puncture were transferred to the test microplates and mixed swiftly. The plates were then incubated at 19°C for 40 h. At the end of the incubation, the plate contents was mixed and 10 µL of cell suspension from each well were withdrawn, diluted with serum-free medium at a ratio of 1:25–1:50 and transferred to a fresh 96-well microplate. This dilution step allowed to obtain—after cell settlement—a RBC monolayer, allowing accurate microscopic counts. GFP-expressing zygotes and ookinetes were visualized using a fluorescence microscope (400× magnification) (Fig. 4) and quantified with the help of an ocular grid. ESS counts were performed in a series of fields along the well diameter. Percent inhibition of ESS development was calculated as follows:

$${\text{Percent inhibition of ESS development }} = \left( {1 - \frac{\text{Mean ESS count in test wells}}{\text{Mean ESS count in solvent control wells}}} \right) \times 100$$

Each substance was tested in 3–6 replicate wells and experiments were repeated at least twice.

Extracts/molecules demonstrating a >80% inhibition at 50 µg/mL were dose ranged to determine the IC_50_ values.

### Evaluation of effects on exflagellation of male gametocytes in vitro

Extracts/molecules found active in the ODA were tested in an exflagellation assay to assess stage-specific effects on male gametogenesis. The exflagellation assay was carried out according to the protocol illustrated above [55, 56]. Mice were treated with phenylhydrazine and infected with *P. berghei* ANKA. On day 4 post-infection, 5 μL tail blood were collected and resuspended in 140 μL exflagellation medium containing 50 μg/mL Ver-EtOH or 50 μM vernodalol, dissolved in DMSO to a maximum DMSO concentration of 0.2%. DMSO and azadirachtin A were used as negative and positive controls, respectively [14]. The number of exflagellation centres per 1,000 RBCs, as an index of microgametogenesis [57, 58], were counted under the microscope (400× magnification), 20 min after mounting the slide chambers. For each concentration of test extract/molecule triplicate slides were counted (Fig. 3).

### Assessment of effects on the feeding capacity of *Anopheles stephensi*

The impact of Ver-EtOH on *An. stephensi* blood feeding was assessed to evaluate whether a reduction in mosquito infection in the in vivo transmission blocking experiment might be the result of decreased blood intake and hence ingestion of a reduced number of gametocytes. Feeding capacity was estimated by measuring the haematin content in the rectal fluid excreted by female mosquitoes during blood feeding. Briefly, narcotized mice, treated with 100 mg/kg extract or solvent control (i.p.) 1 h before, were placed on top of mosquito cages and the rectal fluid from feeding mosquitoes was collected in plastic Petri dishes (15 cm diameter), previously placed inside the mosquito cage. The content of each Petri dish was then dissolved in 20 mL of 0.1 M NaOH and incubated overnight to allow for the conversion of haemoglobin to haematin [59]. To remove debris, the mixture was centrifuged at 13,000 rpm for 10 min. Supernatant samples were then serially diluted twofold in a 96-well microplate and the absorbance was measured at 392 nm wavelength using a FLUOstar Omega microplate reader (BMG Labtech, Germany). Commercial haemin (Sigma-Aldrich) was dissolved in 0.1 M NaOH in order to derive haematin solutions [60] for the preparation of a calibration curve. The concentration of haematin in the diluted samples was then determined by extrapolating the optical density values onto the standard curve.

### Cytotoxicity assessment

General cell toxic effects of *V. amygdalina* constituents were assessed on human colon carcinoma cell line HCT116 and human endothelial cell line (EA.hy926). HCT116 was cultured in RPMI1640 medium with 2 mM l-glutamine, 100 IU/mL penicillin, 100 μg/mL streptomycin, and supplemented with 10% heat-inactivated foetal bovine serum (HI-FBS; PAA Laboratories GmbH, Austria). EA.hy926 was cultured in Dulbecco’s Modified Eagle’s Medium (DMEM) with 2 mM l-glutamine, 100 IU/mL penicillin, 100 µg/mL streptomycin and supplemented with 10% HI-FBS. Cells were cultured in a humidified atmosphere at 37°C in presence of 5% CO_2_.

The cytotoxic activity of extracts, fractions and pure compounds from *V.amygdalina* leaves was measured by the 3-(4,5-dimethylthiazol-2-yl)-2,5-diphenyltetrazolium bromide (MTT) assay [61]. Briefly, 100 µL aliquots of cell suspensions were plated at a density of 2 × 10^4^ cells/mL in flat-bottomed 96-well microplates (Falcon, Becton Dickinson Labware, USA). After 24 h, cells were exposed to compounds, fractions and extracts at concentrations ranging from 0.78 to 200 μg/mL, or 0.1% DMSO as control. Cell cultures were incubated at standard conditions for 72 h. At the end of the incubation, 10 µL/well of MTT (5 mg/mL in phosphate-buffered saline) were added to the plates, and these were further incubated for 4 h at 37°C. Formazan crystals, produced by viable cells, were dissolved in 100 μL of DMSO after removal of the medium from the wells. Absorbance was measured at 540 nm using a FLUOstar Omega microplate reader. Cytotoxicity was expressed as the concentration of extracts, fractions or pure compounds inhibiting cell growth by 50% (IC_50_). The selectivity index (SI) was derived by dividing the IC_50_ value for mammalian cells by that obtained in the ODA against ESS.

### Assessment of transmission blocking activity on *Plasmodium falciparum* field isolates

#### Study site and recruitment of gametocyte carriers

The study was conducted in the area of Bobo-Dioulasso (Southwest Burkina Faso) during the high transmission season (September to October) in 2013 [15]. Gametocyte positive blood samples for the direct membrane-feeding assay (DMFA) were obtained from children aged 5–11 years. Totally 875 children were examined during seven screening events. At each survey the children were clinically examined for the presence of chronic diseases and acute infections including signs of severe malaria. Finger-prick blood was collected for the preparation of thick smears and measurement of haemoglobin using Hemocue (AB Leo Diagnostics, Helsingborg, Sweden). Medication history, namely use of anti-malarial drugs during the last two weeks and hypersensitivity history, was recorded.

Giemsa-stained thick smears were examined at the laboratory of the “Institut de Recherche en Sciences de la Santé” (IRSS) on the same day. On each slide 100 fields were screened for the presence of *Plasmodium* parasites. On positive slides, asexual parasite and gametocyte numbers were determined per 200 and 1,000 leukocytes, respectively. Gametocytaemia and parasitaemia were then expressed as gametocytes/µL and total number of parasites/µL of blood, referring to 8,000 leukocytes/µL of blood [15]. Asymptomatic children with *P. falciparum* gametocytaemia ≥40 gametocytes/µL, parasitaemia ≤1,200 parasites/µL and negative for other *Plasmodium* species were selected as blood donors for the DMFA scheduled for the following day.

All parasitologically confirmed malaria cases during screening for the identification of gametocyte donors were treated with a combination of artesunate (4 mg/kg body weight) and amodiaquine (10 mg/kg body weight), once daily for 3 days.

#### Test fractions

Fractions 11, 13, and 14 from the EtOAc phase were selected based on anti-ESS activity obtained in the ODA with *P.*  *berghei* and were tested for transmission-blocking activity on *P.* *falciparum* isolates.

#### Direct membrane feeding assay

The assay involving *P.* *falciparum* gametocytaemic blood from volunteer individuals and *An. coluzzii* mosquitoes [15] has been validated by Bousema and colleagues for the assessment of transmission blocking interventions [62]. Batches of about 50 female *An.* *coluzzii* mosquitoes were housed in cardboard cups covered with mesh nettings. Aliquots of 500 μL gametocytaemic blood containing fractions 11, 13 and 14 from *V.* *amygdalina* at a concentration of 100 μg/mL were placed in glass feeders and mosquitoes were allowed to feed for 45 min. Unfed mosquitoes were discarded. On day 7 after membrane feeding, all surviving mosquitoes were examined for prevalence and density of oocysts. Mosquito midguts were dissected and mounted in PBS containing 1% mercurochrome as an oocyst stain. Geometric means of oocyst densities [53] were calculated considering positive mosquitoes only.

### Ethical clearance

The field study involving venous blood collection from children infected with *P.* *falciparum* was approved by the Ethical Committee of the Centre Muraz and filed under the registration number N/Ref. 003-2009/CE-CM. Parents or guardians provided written informed consent before children were enrolled into the blood sample collection.

The experiments conducted with mice in the animal rearing facilities of the University of Camerino had been examined by the Ethical Committee of the University (*COMITATO ETICO DI ATENEO* Protezione degli Animali utilizzati a fini sperimentali o altri fini scientifici CEAPA) to be compliant with the Italian Legislative Decree on the “use and protection of laboratory animals” (D. Lgs. 116 of 10/27/92, paragraph 7) and had been approved by the Counsel of the School of Pharmacy, University of Camerino.

### Statistical analyses

Excel 2007 spreadsheet (Microsoft office) and GraphPad Prism 6 statistical software (GraphPad Software, San Diego, CA, USA) were used for data analysis. Normally distributed data, namely gametocyte densities, haematin values, ESS counts and derived % inhibition values, were expressed as arithmetic means and 95% confidence intervals (95% CI), whereas oocyst densities among infected mosquitoes were expressed as geometric means and 95% CI. The independent samples Student’s *t*-test was used to compare means and Fisher’s exact test to compare categorical data. The a priori statistical significance level (α) was set at 0.05.

## Results

### Gametocytocidal activity in vivo

On day 7 after exposure to infectious mosquito bites, mice treated orally with 500 mg/kg Ver-H_2_O for 9 days showed a 46.8 and 45.4% reduction in macrogametocyte densities in two independent experiments. In the first one, mean counts of 48 and 25.5 macrogametocytes/2,500 infected red blood cells (iRBCs) were recorded in control and treatment mice, respectively, and respective counts of 42.7 and 23.3 (p < 0.05) were obtained in the replicate experiment. Ver-H_2_O appeared to affect also microgametocytes but a significant reduction of male sexual forms (p < 0.05) was observed only in one of the two experiments. Ver-EtOH treatment at 500 mg/kg did not impact significantly on macro- or microgametocyte densities (Table 1).

**Table 1:** Effect of *Vernonia amygdalina* leaf extracts on *Plasmodium berghei* gametocyte densities

Treatment groups	Experiment^a^	Microgametocytes per 2,500 IRBCs (95% CI)	Macrogametocyte per 2,500 IRBCs (95% CI)
Ver-EtOH	1	6.7 (5.4–8.0)	40.3 (35.9–44.7)
	2	6.8 (5.6–8.0)	38.4 (32–44.8)
Ver-H_2_O	1	5.5 (4.9–6.1)	25.5 (22.8–28.2)*
	2	5.3 (4.9–5.7)*	23.3 (20.7–26.0)*
Control	1	7.2 (5.1–9.3)	48 (34.7–61.3)
	2	8.5 (6.1–10.9)	41.7 (33.6–49.7)

Data are presented as arithmetic means with 95% confidence intervals (95% CI).

*IRBCs* infected red blood cells, *Ver-EtOH* ethanolic *V.* *amygdalina* leaf extract, *Ver-H*_*2*_ *O* aqueous *V.* *amygdalina* leaf extract *p < 0.05 with respect to their control.

^a^Numbers 1 and 2 refer to the two replicate experiments each involving treatment and control groups consisting of six mice each.

Examining the same slides of the two experiments for asexual forms, Ver-EtOH was found to reduce parasitaemia by 59.2% (95% CI 52.6–65.7%). Parasitaemia in the groups treated with Ver-EtOH amounted 3.6 and 8.7% in the control groups (p < 0.05). Ver-H_2_O reduced parasitaemia by 33.6% (95% CI 26.7–40.2%), parasitaemia values of 5.8 and 8.7% were recorded in the treatment and control groups, respectively (p < 0.05).

### Transmission blocking activity in vivo

Both, Ver-EtOH and Ver-H_2_O, when administered to mosquitoes through a blood meal on treated gametocytaemic mice, impacted on the vector infection. Ver-H_2_O reduced oocyst density by 54.7% (95% CI 35.3–72.1). The mean number of oocysts amounted 266 in control mosquitoes fed on solvent-treated mice and 123 in the mosquitoes fed on mice treated with Ver-H_2_O (p < 0.05). The number of uninfected mosquitoes (infection prevalence) was similar in Ver-H_2_O and control mosquitoes (Table 2). Ver-EtOH reduced both infection prevalence and intensity. The prevalence amounted 71.4% (95% CI 61.3–81.5%) in mosquitoes fed on Ver-EtOH, compared with 97.6% (95% CI 94.4–100.9%) in controls, corresponding to a significant reduction of 27% (p < 0.05). Among the infected mosquitoes, the mean oocyst density was 374 in control mosquitoes versus 37 in Ver-EtOH treated mosquitoes, resulting in a 90% reduction of oocyst numbers (95% CI 83.2–96.8%; p < 0.05) (Table 2).

**Table 2:** Effect of ethanolic and aqueous leaf extracts of *Vernonia amygdalina* on *Plasmodium berghei* oocyst development in *Anopheles* *stephensi* mosquitoes

Group	Mouse ID^a^	Prevalence of infected mosquitoes (infected/total examined)	Oocyst density by mouse replicate (95% CI)	Oocyst density by treatment group (95% CI)
Ver-EtOH	1	76.9 (20/26)	27 (13–57)	37 (26–53)*
	2	29.4 (5/17)	12 (5–28)	
	3	88.2 (30/34)	56 (32–96)	
Sol-EtOH	4	100 (34/34)	417 (297–585)	374 (297–467)
	5	96.4 (27/28)	439 (319–605)	
	6	95.7 (22/23)	260 (152–447)	
Ver-H_2_O	7	100 (20/20)	72 (45–103)	123 (83–149)*
	8	100 (20/20)	151 (101–161)	
	9	100 (20/20)	143 (104–182)	
Sol-H_2_O	10	100 (20/20)	272 (225–319)	266 (200–300)
	11	100 (20/20)	242 (186–298)	
	12	100 (20/20)	237 (190–284)	

The oocyst densities (geometric mean of oocysts/mosquito) were calculated on oocyst positive mosquitoes only.

*Ver-EtOH* ethanolic *V.* *amygdalina* leaf extract, *Ver-H*_*2*_ *O* aqueous *V.* *amygdalina* leaf extract, Sol-EtOH and Sol-H_2_O are solvent controls for the ethanolic and aqueous extract, respectively.

* Oocyst density in extract treated groups significantly different with respect to the solvent controls; P < 0.05.

^a^Each number represents one gametocytaemic treatment or control mouse used for the infection of a separate batch of mosquitoes (100–150 females per mouse).

In Ver-EtOH experiments, the feeding capacity of treatment and control mosquitoes was estimated by measuring the amount of haematin excreted with the rectal fluid. Mosquitoes offered a blood meal on mice treated with Ver-EtOH took slightly but not significantly less blood compared with mosquitoes fed on control mice. The mean amount of haematin was 5 µg per mosquito (95% CI 4.34–5.66 µg) in those having fed on Ver-EtOH treated mice versus 6.2 µg per mosquito (95% CI 5.2–7.2 µg) in the control group (p = 0.13). Similar results were obtained in a replicate experiment, yielding a value of 8.6 µg per fed mosquito (95% CI 7.53–9.67 µg) in the extract-exposed group versus 11.1 µg per mosquito (95% CI 9.6–12.6 µg) in controls (p = 0.06). Five microgram approximately corresponds to the haematin content of 1 µL blood.

### Impact on the development of early sporogonic stages in vitro

#### Ethanolic and aqueous extracts

The two extracts found active in our in vivo transmission blocking study (Ver-EtOH and Ver-H_2_O) were investigated in vitro for inhibition of ESS, hypothesizing that the ESS developing in the mosquito midgut in the first 24 h after blood ingestion, are likely targets for the bioactive extract metabolites. Ver-EtOH tested at the initial screening dose of 50 μg/mL was found to inhibit ESS development by 81.3–96.2%, while the aqueous extract showed no inhibitory activity (Table 3).

**Table 3:** Impact of *Vernonia amygdalina* leaf extracts on the development of early sporogonic stages in vitro

Test agents	Experiment^a^	Mean ESS counts (95% CI)^b^	Percentage inhibition of ESS (95% CI)
Ver-EtOH	1	53 (46–60)	81.3 (79.1–83.7)
	2	11 (3–19)	96.2 (93.4–99.1)
	3	18 (15–20)	87.5 (85.8–89,14)
	4	45 (41–50)	88 (86.7–89.4)
Sol-EtOH	1	285 (239–332)	
	2	283 (261–306)	
	3	141 (120–163)	
	4	378 (310–446)	
Ver-H_2_O	5	51 (42–60)	14 (5–23.1)
	6	23 (14–32)	38.7 (14.2–63.3)
Sol-H_2_O	5	59 (51–68)	
	6	37 (32–42)	

Ver-EtOH and Ver-H_2_O were tested at 50 µg/mL.

Data are presented as mean early sporogonic stages (ESS) counts calculated from triplicate wells and percentages of ESS inhibition referred to solvent controls. Both parameters are presented with 95% confidence intervals (95% CI).

*Ver-EtOH* ethanolic *V.* *amygdalina* leaf extract, *Ver-H*_*2*_ *O* aqueous *V.* *amygdalina* leaf extract, Sol-EtOH (dimethyl sulfoxide at 0.2%) and Sol-H_2_O (phosphate buffered saline) are solvent controls for the ethanolic and aqueous extract, respectively.

^a^Each number represents one experiment conducted with gametocytaemic blood from a different mouse.

^b^Mean ESS counts are rounded to the nearest whole numbers.

**Fig. 5:**
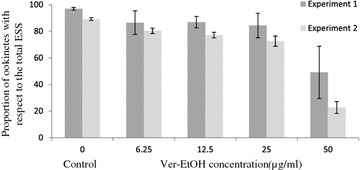
Proportion of mature ookinetes with respect to total early sporogonic stages. The test was conducted using ethanolic *Vernonia* *amygdalina* leaf extract (Ver-EtOH) at different concentration and solvent control (dimethyl sulfoxide). *Each bar* in the graph refers to readings from 3 and 6 microplate wells in experiment 1 and 2, respectively.

Dose range experiments performed with Ver-EtOH allowed us to determine an IC_50_ value of 15.4 µg/mL (95% CI 12.7–18.8 µg/mL). Enumerating zygotes and ookinetes separately, the proportion of ookinetes, which include zygotes initiating elongation and developing to fully mature (“banana shaped”) ookinetes, was found to be reduced at the highest Ver-EtOH test concentration. In control wells 90–98 % of zygotes were recorded to undergo ookinete development, whereas in those to which Ver-EtOH was added at50 µg/mL only about one fifth to one half of total ESS presented elongated forms (Fig. 5).

#### Phases and fractions

Following the congruent results emerging from the in vitro and in vivo transmission blocking experiments with the Ver-EtOH, bioassay guided fractionation studies were undertaken to identify the molecule(s) responsible for the observed activity against EES.

Ver-MeOH (for the procedures of chemical analysis, MeOH was preferred to EtOH as extraction solvent, since they have practically identical extracting efficiency) revealed moderate in vitro activity against ESS, at the initial screening dose of 50 µg/mL, consisting in a 51–68.3% ESS development inhibition (Table 4). The BuOH phase separated from Ver-MeOH displayed higher inhibitory activity (>86%). This fraction, however, was found to be cytotoxic to RBCs provoking evident haemolysis at this dosage. The EtOAc phase displayed an inhibition range of 58.3–79.1%, whereas the water phase showed poorly reproducible effects (Table 4). Based on these results, the EtOAc phase was selected for further fractionation, whereas the BuOH and H_2_O phases were not considered for further study due to cytotoxicity, for the former, and doubts on the specificity of the effects displayed by the latter. The EtOAc phase was subjected to medium pressure liquid chromatography (MPLC) and 14 fractions were obtained. Based on proton-NMR preliminary analysis, four fractions containing fatty acids and/or triglycerides were not considered for testing, while ten fractions were screened in vitro for impact on ESS development. Fractions 2, 3, 5, 8, 9, and 10 did not exhibit relevant reproducible activity at 50 μg/mL, whereas fractions 11 to 14 caused almost complete ESS inhibition (>90%). These fractions were eluted with mobile phases of different polarity, namely fr 11 [*n*-hexane:EtOAc (1:1 to 7:11)], fr 12 [*n*-hexane:EtOAc (7:11 to 1:4)], fr 13 [EtOAc] and fr 14 [EtOAc:MeOH (1:1)] (Table 5).

**Table 4:** Impact of *Vernonia amygdalina* leaf methanolic extract and derived phases on the development of early sporogonic stages

Test agents	Experiment^a^	Mean ESS (95% CI)^b^	Percentage inhibition of ESS (95% CI)
Ver-MeOH	1	13 (9–18)	51.0 (34.2–67.8)
	2	4 (3–6)	68.3 (58.7–77.9)
	3	41 (34–47)	57.5 (50.6–64.4)
	4	105 (92–118)	51.9 (45.9–57.9)
	5	57 (47–66)	57.9 (50.9–64.9)
Phases from methanolic extract			
Water	1	13 (7–20)	51.0 (26.6–75.4)
	2	5 (2–7)	65.9 (46.7–85.0)
	3	8 (0–8)	91.6 (86.2–97.1)
	4	115 (76–154)	47.5 (29.8–65.1)
	5	76 (59–93)	43.6 (31–56.1)
Ethylacetate	1	11 (10–13)	58.3 (52.0–64.7)
	2	4 (2–5)	73.2 (60.5–85.8)
	3	20 (15–25)	79.1 (73.7–84.5)
	4	76 (40–112)	65.4 (49.0–81.9)
	5	48 (37–59)	64.1 (55.9–72.3)
Butanol	1	1 (0–3)	95.1 (88.7–100)
	2	1 (1–2)	90.2 (85.5–95.0)
	3	2 (1–4)	97.6 (95.8–99.4)
	4	15 (7–22)	93.3 (89.9–96.7)
	5	18 (14–23)	86.4 (82.9–89.9)
Solvent control (0.2% DMSO)	1	27 (22–33)	
	2	14 (9–19)	
	3	96 (85–106)	
	4	219 (198–240)	
	5	135 (111–158)	

Methanolic *V. amygdalina* leaf extract and its butanol, ethylacetate and water phases were tested at 50 µg/mL.

Data are presented as mean counts of early sporogonic stages (ESS) calculated from triplicate wells and percentages of ESS inhibition referred to solvent control. Both parameters are presented with 95% confidence intervals (95% CI).

*Ver-MeOH* methanolic extract of *V.* *amygdalina* leaves.

^a^Each number represents one experiment conducted with gametocytaemic blood from a different mouse.

^b^Mean ESS counts are rounded to the nearest whole number.

**Table 5:** Inhibitory activity of ethylacetate phase fractions from *Vernonia amygdalina* leaves against early sporogonic stages

Test agents	Experiment^a^	Mean ESS counts (95% CI)^b^	Percentage inhibition of ESS (95% CI)
Fraction-2	1	27 (17–37)	0.7 (0–38.2)
	3	80 (70–90)	16.0 (5.7–26.4)
	4	221 (159–281)	0 (0–27.5)
Fraction-3	1	27 (20–33)	2.0 (0–25.6)
	3	93 (77–109)	2.8 (0–19.8)
	4	186 (128–245)	14.9 (0–41.7)
Fraction-5	1	29 (25–33)	0.0 (0–6.8)
	3	40 (25–56)	57.8 (42.0–73.7)
	4	215 (149–281)	1.8 (0–32.1)
Fraction-7	1	23 (13–33)	15.4 (0–51.0)
	3	98 (84–113)	0 (0–12.2)
	4	224 (211–238)	0 (0–3.7)
Fraction-9	1	31 (19–42)	0.0 (0–29.7)
	3	57 (51–63)	40.4 (34.2–46.7)
	4	195 (160–229)	11.1 (0–26.8)
Fraction-10	1	19 (10–27)	31.4 (0–63.7)
	2	6 (4–8)	56.1 (26.2–86.0)
	3	71 (57–84)	26.3 (12.5–40.1)
	4	187 (161–213)	14.8 (3.0–26.6)
Fraction-11	1	1 (0–2)	96.3 (92.2–100)
	2	0	100
	3	0 (0–1)	99.7 (99–100)
	4	1 (0–1)	99.7 (99.4–100)
	5	0	100
Fraction-12	1	0	100
	2	1 (0–2)	95.1 (85.6–100)
	3	0 (0–1)	99.7 (99–100)
	4	1 (0–2)	99.7 (99.1–100)
	5	0 (0–1)	99.8 (99.3–100)
Fraction-13	1	0	100
	2	0 (0–1)	97.6 (92.8–100)
	3	0	100
	4	0 (0–1)	99.8 (99.5–100)
	5	1 (0–2)	99.5 (98.5–100)
Fraction-14	1	0	100
	2	1 (0–2)	92.7 (84.4–100)
	3	0	100
	4	0	100
	5	0	100
Solvent control (0.2% DMSO)	1	27 (22–33)	
	2	14 (9–19)	
	3	96 (85–106)	
	4	219 (198–240)	
	5	135 (111–158)	

Fractions from ethylacetate phase were tested at 50 µg/mL concentration.

Data are presented as mean early sporogonic stages (ESS) counts calculated from triplicate wells and percentages of ESS inhibition referred to solvent control. Both parameters are presented with 95% confidence intervals (95% CI.)

^a^Each number represents one experiment conducted with gametocytaemic blood from a different mouse.

^b^Mean ESS counts are rounded to the nearest whole number.

### Transmission blocking activity of *Vernoniaamygdalina* leaf fractions against *Plasmodium falciparum* field isolates

Fractions demonstrating activity against *P. berghei* ESS in the ookinete development assay were tested for confirmation of transmission blocking activity on field isolates of the human malaria parasite *P.* *falciparum*. All fractions affected the sporogonic development of *P.* *falciparum* in *An.* *coluzzii* mosquitoes when administered to females by membrane feeding, at 100 µg/mL.

**Fig. 6:**
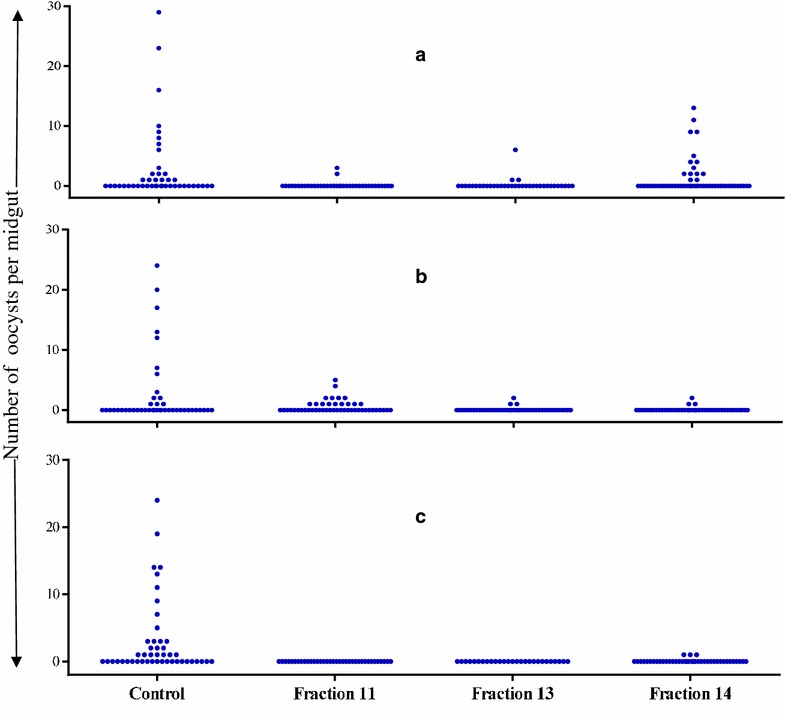
Impact of organic solvent fractions from *Vernonia amygdalina* leaves (100 µg/mL) on sporogonic development. Mosquitoes were membrane fed on blood from three donors with *Plasmodium* *falciparum* gametocytaemia values of: **a** 160 gametocytes/μL, **b** 184 gametocytes/μL and **c** 80 gametocytes/μL. *Each point* represents the oocyst number from an individual mosquito. In **a** the data point of one midgut on which 94 oocysts were recorded, is not plotted in the graph (largely out of scale).

The number of infected mosquitoes was reduced upon treatment with fraction 11, 13 and 14, however prevalence values varied conspicuously across replicate experiments (Table 6). In control mosquitoes fed on DMSO-supplemented gametocytaemic blood, oocyst prevalence ranged from 30 to 50%. Fraction 13 was found to block mosquito infection in one replicate experiment and to significantly reduce oocyst prevalence to 6.5 and 8.3% in the other two. A significant reduction in oocyst prevalence was also recorded with fraction 11 and 14 in two out of the three replicate experiments. Fraction 11 inhibited oocyst development completely in one occasion (Table 6; Fig. 6). Oocyst density, assessed on positive midguts only, was reduced overall by about a half in treated mosquitoes (Table 6; Fig. 6).

**Table 6:** Transmission blocking activity of ethylacetate fractions from *Vernonia amygdalina* leaves on *Plasmodium* *falciparum* isolates

Blood sample	Gametocytes per µL blood	Treatment	% Prevalence of infected mosquitoes (infected/total examined)	Oocyst density^a^ (95% CI)
1	160	Solvent control	42.2 (19/45)	4.2 (2.7–6.6)
		Fraction-11	5.1 (2/39)*	2.4 (1.6–3.6)
		Fraction-13	8.3 (3/36)*	1.8 (0.6–5.9)
		Fraction-14	26.2 (11/42)	3.5 (2.3–5.5)
2	184	Solvent control	29.6 (13/44)	3.6 (1.7–5.5)
		Fraction-11	32.4 (12/37)	1.8 (0.3–3.3)
		Fraction-13	6.5 (3/46)*	1.3 (0–2.8)
		Fraction-14	6.7 (3/45)*	1.3 (0–2.8)
3	80	Solvent control	50 (23/46)	3.5 (2.2–5.4)
		Fraction-11	0 (0/36)*	–
		Fraction-13	0 (0/27)*	–
		Fraction-14	7.3 (3/41)*	1

Fisher’s exact test was used to compare the proportion of infected mosquitoes between control and treatment groups.

^a^Oocyst density was calculated considering infected mosquitoes only. *P < 0.05 with respect to the solvent control (dimethyl sulfoxide). Fractions were tested at 100 µg/mL.

### Activity of pure compounds from *Vernonia* *amygdalina* molecules against *Plasmodium berghei* early sporogonic stages

HPLC purification and subsequent NMR-based structural characterization of the constituents of the active fractions allowed the identification of the sesquiterpene lactone vernolide as the major compound of fraction 11 and 12 and vernodalol (Fig. 1) of fractions 13 and 14. The two compounds were tested in the ODA at 50 μM, revealing vernodalol as the most active molecule, inhibiting ESS development by 70–90%. Vernolide showed weak and variable inhibitory activity (Table 7). Dose range experiments conducted with vernodalol allowed to determine an IC_50_ value of 18.7 µM (95% CI 16.1–21.7 µM). Evaluating numbers of zygotes and ookinetes separately, the proportion of zygotes initiating elongation or developing to banana shaped mature ookinetes did not appear to be affected by vernolide, vernodalol or the fractions rich in the two molecules (Additional file 1: Table S1, Additional file 2: Table S2).

**Table 7:** Impact of vernolide and vernodalol on the development of early sporogonic stages in vitro

Test compounds	Experiment^a^	Mean ESS counts (95% CI)^b^	Percentage inhibition of ESS (95% CI)
Vernodalol	1	24 (21–26)	79.3 (77.3–81.4)
	2	12 (8–16)	89.7 (86.4–92.9)
	3	92 (77–107)	69.3 (64.3–74.3)
Vernolide	1	103 (90–115)	9.4 (0–20.5)
	2	79 (63–95)	30.6 (16.7–44.5)
	3	201 (182–220)	33 (26.6–39.3)
Solvent control (0.2% DMSO)	1	113 (93–134)	
	2	114 (97–132)	
	3	300 (284–317)	

Compounds were tested at a concentration of 50 μM.

^a^Each number represents one experiment conducted with gametocytaemic blood from a different mouse; ESS counts are means from sextuplicate wells.

^b^Mean ESS counts are rounded to the nearest whole numbers.

### Effect of vernodalol and ethanolic *Vernonia* *amygdalina* leaf extract on microgamete formation

Vernodalol and Ver-EtOH were tested in the exflagellation assay to assess whether the effect observed on early sporogonic development in the ODA was due to a specific action on microgametogenesis. Vernodalol tested at 50 μM and Ver-EtOH at 50 μg/mL demonstrated high inhibitory activity on ESS (Tables 5, 8), but did not influence microgamete formation. Similar numbers of exflagellation centres per 1,000 RBCs were observed, namely 5.5 (95% CI 4–7), 4.8 (95% CI 0–11) and 4.6 (95% CI 2.3–6.8), for the solvent control, vernodalol (50 μM) and Ver-EtOH (50 μg/mL), respectively. For comparison, exflagellation centres counts for azadirachtin A used as a positive control in the assay at 50 μM amounted 0.29 (95% CI 0–0.67).

### Cytotoxicity of *Vernonia* *amygdalina* extracts, fractions and isolated compounds

Ver-EtOH and Ver-MeOH demonstrated a relatively higher cytotoxic effect compared with Ver-H_2_O. Fractions active against ESS development, in particular the vernolide-rich fraction 11 and 12, showed strong growth inhibitory effects on both cell lines, HCT116 and EA.hy 926 (Table 8). The selective index (SI) values (ratio of the 50% inhibitory concentration of mammalian cell viability and of ESS growth) of Ver-EtOH (9.46 μg/mL:15.4 μg/mL) and vernodalol (7.1 µM:18.7 µM) were found to be less than one.

**Table 8:** Activity of *Vernonia* *amygdalina* extracts, fractions and pure compounds on cell viability of a human colon cancer and endothelial cell line

Test agents	IC_50_ (95% CI)	
	HCT116	EA.hy 926
Leaf extracts (μg/mL)		
Ver-EtOH	3.66 (3.41–3.96)	9.46 (8.64–10.36)
Ver-MeOH	8.09 (7.54–8.67)	16.94 (15.55–18.46)
Ver-H_2_O	64.34 (58.75–70.53)	76.21 (58.09–100.00)
Fractions from methanol extract (μg/mL)		
Fraction 11	0.17 (0.15–0.18)	0.42 (0.39–0.45)
Fraction 12	0.33 (0.3–0.37)	1.13 (0.97–1.31)
Fraction 13	2.41 (2.29–2.53)	4.52 (4.23–4.82)
Fraction 14	3.86 (3.62–4.12)	7.69 (7.10–8.32)
Molecules from fractions (μM)		
Vernolide	1.49 (1.38–1.61)	3.18 (2.97–3.4)
Vernodalol	4.97 (4.78–5.17)	7.1 (6.51–7.74)

Each value represents the mean of two experiments carried out in quadruplet wells.

Ver-EtOH, Ver-MeOH, and Ver-H_2_O stands for ethanolic, methanolic and aqueous extract of *V.* *amygdalina* leaves.

*HCT 116* human colon carcinoma cell line, *EA.hy 926* human (non-tumour) endothelial cell line, *95% CI* 95% confidence interval.

## Discussion

Exploratory in vivo studies conducted with *P. berghei* ANKA strain and *An. stephensi* mosquitoes revealed transmission blocking activity of *V.amygdalina* aqueous (Ver-H_2_O) and ethanolic (Ver-EtOH) leaf extracts. *Anopheles stephensi* females, fed on gametocytaemic mice treated with Ver-H_2_O at 500 mg/kg and Ver-EtOH at 100 mg/kg, developed respectively 55 and 90% fewer oocysts compared to controls. Subsequent fractionation studies, guided by in vitro assays, revealed activity against the early stages of sporogonic development in four fractions of the ethylacetate phase. Chemical analysis allowed isolation and identification of the two compounds most likely responsible for the transmission blocking activity observed, namely vernodalol and vernolide. These two molecules are sesquiterpene lactones belonging to the structural classes of germacranolides (vernolide) and elemanolides (vernodalol).

Tested as isolated pure compounds, vernodalol and vernolide exhibited moderate to low activity in the in vitro ookinete development assay (ODA). At a concentration of 50 μM (19.6 μg/mL) vernodalol inhibited early sporogonic development (ESS) by 70–90%, while vernolide at the same concentration (50 μM, 18.1 μg/mL) reduced ESS by 9–33%. In line with these data, *V.* *amygdalina* EtOAc fractions composed of almost only vernodalol and vernolide showed evident inhibitory activity in the ODA. Fraction 11 (about 91% vernolide), fraction 12 (about 50% vernolide + 50% vernodalol) and fraction 13 (about 85% vernodalol) suppressed ESS by 95–100% when tested at 50 μg/mL (corresponding to amounts of 42–50 μg/mL of vernolide, vernodalol or vernolide + vernodalol).

A relatively stronger activity than expected for its vernodalol and vernolide content, was observed with the EtOAc phase preparation. At 50 μg/mL, corresponding to 2.04 μg/mL vernolide and 2.16 μg/mL vernodalol equivalents, the preparation inhibited ESS by 60–80%. This finding may indicate the presence of other bioactive constituents in the EtOAc phase, having a direct effect on ESS or modulating the activity of vernolide and vernodalol. The same interpretation may hold for the results obtained with Ver-EtOH, which was found to inhibit ESS by 80–95% at 50 μg/mL but contained vernolide and vernodalol at a low concentration of 0.84 and 0.89 μg/mL, respectively. Differential counting of zygotes and ookinetes indicated that Ver-EtOH interfered with processes preceding zygote formation. At the highest tested concentration (50 μg/mL), however, an impact on subsequent ookinete maturation was also observed. Interestingly, this was not the case with pure vernolide and vernodalol or with fractions 11 to 13, consisting almost entirely of these two compounds, an observation suggesting that the two sesquiterpene lactones may interfere with the first processes of sporogonic development, namely gametogenesis and/or macrogamete fertilization.

Exflagellation tests performed with vernodalol and Ver-EtOH allowed to further narrow down the possible target stages. Both the compound and the extract, tested at 50 μM and 50 μg/mL respectively, did not interfere with microgamete formation in vitro, suggesting that the processes targeted by *V.amygdalina* transmission blocking components are mainly macrogametogenesis and/or macrogamete fertilization.

Vernolide and vernodalol have been reported to exhibit moderate activity also on asexual blood stages (39, 40), inhibiting *P. falciparum* asexual forms by 50% at a concentration of 1.87–8.4 and 4.2 μM, respectively. This multi-stage activity of vernodalol and vernolide raises exciting questions on modes and mechanisms of action of the two sesquiterpene lactones and research focused into this aspect may lead to the discovery of novel multistage drug targets.

In our exploratory in vivo transmission blocking experiment, Ver-EtOH was found to reduce the prevalence of infection in mosquitoes fed on gametocytaemic mice treated (i.p.) with the extract at 100 mg/kg by about 30%, and oocyst density by 90%. Taking into account the relative amount of vernolide and vernodalol in Ver-EtOH, at the tested extract dosage mice received approximately 1.7 mg/kg vernolide and about 1.8 mg/kg vernodalol. Given this relatively low doses of the transmission blocking compounds, other bioactive molecules in the extract are likely to have contributed directly or indirectly to the transmission blocking effect, as discussed also above relating to Ver-EtOH activity on early sporogonic development in vitro.

The experiments conducted in Burkina Faso with *P.falciparum* field isolates allowed us to evidence the transmission blocking activity of *V. amygdalina* also against the human malaria parasite. *An.coluzzii* mosquitoes that had membrane-fed on gametocytaemic blood treated with the vernolide and vernodalol-rich EtOAc fractions (fraction 11 and 13, respectively) were less infected and oocyst numbers in positive mosquitoes were reduced by about half. The membrane feeds were supplemented with the fractions at a dosage of 100 μg/mL corresponding to vernolide and vernodalol equivalents of 91.2 and 85 μg/mL, respectively. The relatively high dosage needed to achieve a substantial reduction in sporogonic development of *P. falciparum*, taken together with the moderate activity by the pure compounds observed in vitro on *P.berghei*, suggest that neither vernodalol nor vernolide possess the potency required for a drug candidate molecule. On the other hand, significant transmission blocking activity by the Ver-EtOH was evidenced from the in vivo experiments; and the bio-guided fractionation studies revealed a relatively higher in vitro activity of extracts compared to that of the pure compounds, suggesting that the development of a phytomedicine based on vernodalol and vernolide enriched standardized extract may be a more appropriate strategy to pursue.

Artemisinin derivatives have been demonstrated to possess activity against *P. falciparum* gametocytes in vitro [63] and in clinical trials [3], shortening the period of gametocytes circulation in patients treated with ACT [64] and thus decreasing the infection rates of blood seeking mosquitoes. In the current in vivo study, Ver-H_2_O administered to mice at 500 mg/kg was found to reduce macrogametocyte densities by about 50%. A decrease in microgametocyte numbers was also noticed, reaching significance in one of the two replicates carried out. Since Ver-H_2_O did not exhibit in vitro activity on ESS, but still reduced oocyst densities in mosquitoes when administered to females in vivo through an infectious blood meal, the observed transmission blocking effect might be attributed to the gametocytocidal activity of Ver-H_2_O.

Considering the diverging gametocyte biology of *P.falciparum* and *P.berghei* [65], a validation of the extract’s activity on *P.falciparum* gametocytes is the next step to be undertaken. If confirmed, subsequent bio-guided fractionation studies may allow the identification of new gametocytocidal compounds.

*Vernonia amygdalina* leaves have been the object of various studies evidencing the presence of bio-active secondary metabolites belonging to the classes of saponin, flavonoid, alkaloid, tannin, terpenes, phytosteroids [66–69]. From a comparative study on the profile of *V.amygdalina* secondary metabolites of an aqueous and an ethanol extract emerged an abundant presence of polar polyphenols in the former [70]. This feature has been confirmed by other chemical investigations on Ver-H_2_O extracts [67, 68]. In the present study, sesquiterpene lactones, namely vernolide and vernodalol, were identified in the less polar phase of Ver-MeOH extract. Considering the similar polarity index of ethanol and methanol [71], it seems correct to assume that Ver-EtOH used in the present study contains sesquiterpene lactones as well. On the contrary, given their poor water solubility, it is very unlikely that sesquiterpene lactones can be present in Ver-H_2_O [72], thus providing chemical bases to the difference in biological responses between Ver-EtOH and Ver-H_2_O.

The cytotoxic properties of *V.amygdalina* extracts and compounds have been assessed on various cell lines: IC_50_ values vary widely with the compounds and type of cell lines used. For instance, a value of 0.11 μg/mL was estimated for vernolide against leukemia cells [73] and 70–75 μg/mL for vernodalinol against human breast carcinoma cells [33]. Studies assessing in vitro anti-plasmodial and cytotoxic activity estimated SI values in the range of 5–10 for ethanol and hydroethanol extracts [74, 75], while for a chloroform extract, an SI value of less than one was reported [76]. Pedersen and colleagues determined vernodalol IC_50_ value against *P.falciparum* blood stages and on a Chinese hamster ovarian cell line of 3.8 and 26.4 μg/mL, respectively, implying an SI value of seven [77]. In the present study, both, Ver-EtOH and vernodalol, were found to exhibit SI value below one. Referring to the antiplasmodial drug development consensus, a hit compound should display an SI value of at least ten (a tenfold higher activity against parasites than against a mammalian cell line) to be considered for further development [78]. However, given the interesting multi-stage activity of vernodalol and vernolide, the molecules still are worth to be considered for structure–activity relationship studies aimed at designing compounds with reduced toxicity and enhanced activity against ESS and asexual blood stage parasites. Also, the compounds might be utilized as tools to investigate potential drug targets expressed in multiple life cycle stages.

From studies aimed at elucidating the effects of vernolide on the immune response of melanoma-bearing mice and vernodalin (a sesquiterpene related to vernodalol) on antischistosomal activity in cercaria-infected mice, no serious adverse effect was observed in the animals after administration of vernolide at the immune modulatory dose of 0.5 mg/kg i.p. [79] and vernodalin at antischistosomal dose of 2.5 mg p.o [40]. In the present study, mice were treated with Ver-EtOH at 100 mg/kg [vernolide 1.68 mg/kg + vernodalol 1.78 mg/kg equivalent dose] i.p. twice and 500 mg/kg [vernolide 8.4 mg/kg and vernodalol 8.9 mg/kg equivalent dose] p.o. for 9 days in the in vivo efficacy experiments. At these doses the extracts appeared tolerable, with no visible signs of toxic effects on the mice. Information on the median lethal dose (LD_50_) of Ver-EtOH and Ver-H_2_O in rodents is also available from various studies: LD_50_ estimates of Ver-EtOH range from 288 mg/kg (i.p.) in mice to 1,950 mg/kg (p.o.) in rats whereas those for Ver-H_2_O from 560 mg/kg (i.p.) in mice to 3,320 mg/kg (p.o.) in rats [30, 80–84]. A sub-acute toxicity evaluation of Ver-H_2_O in rats showed that the extract did not cause any life-threatening adverse effect up to the dose of 2,000 mg/kg (p.o.) though at a dosage of 1,000 mg/kg a decrease RBC counts, an increased level of bilirubin and uric acid had been recorded [30]. In a sub-chronic toxicity study of an organic solvent fraction from Ver-MeOH (up to 320 mg/kg, p.o.), no clinical chemistry and histological abnormalities in vital organs were observed in rats [85]. These results are in agreement with our observations on the tolerability of *V.* *amygdalina* extracts at the doses administered to mice in the current study for the evaluation of in vivo transmission blocking activity of Ver-EtOH (100 mg/kg i.p. once daily for 2 days) and the gametocytocidal evaluation (250 mg/kg p.o. twice daily for 9 days).

## Conclusions

Previously published work and findings from the present study demonstrate that *V.* *amygdalina* leaves contain molecules affecting multiple stages of *Plasmodium*, evidencing its potential for drug discovery and for the development of standardized *V.* *amygdalina*-based phytomedicines. On the identified hit molecules, primarily vernodalol, chemical modification is recommended to generate a druggable compound with enhanced activity against the blood and sporogonic stages of the malaria parasite and reduced off-target activities. In addition, based on the findings presented here, the development of a multistage phytomedicine, exhibiting both anti-blood stage as well as transmission blocking properties and designed as a preventive treatment to complement existing malaria control tools appears a challenging but feasible goal.

## Additional files

Additional file 1: Table S1. Ratio of ookinete to total early sporogonic stage counts in vernolide and vernodalol treated microplate wells. Additional file 2: Table S2. Ratio of ookinete to total early sporogonic stage counts in microplate wells treated with *Vernonia amygdalina* fractions.
