# Supplementary material for: Plasmodium transmission blocking activities of Vernonia amygdalina extracts and isolated compounds
Source: Malar J. 2015 Jul 25;14:288. doi: 10.1186/s12936-015-0812-2 (PMC4513948; doi:10.1186/s12936-015-0812-2)
Supplement: Additional file 1: Table S1. — Ratio of ookinete to total early sporogonic stage counts in vernolide and vernodalol treated microplate wells. [file 12936_2015_812_MOESM1_ESM.docx]

**Table S1: Ratio of ookinete to total early sporogonic stage counts in vernolide and vernodalol treated microplate wells.**

| **Experiment** | **Test agents** | **Average ESS** | **Average** | **Ookinete/ESS** |
| --- | --- | --- | --- | --- |
|  |  |  | **ookinete** | **(%95 CI)** |
| 1 | DMSO control | 114.3 | 109 | 0.95 (0.94 − 0.97) |
|  | Vernolide | 79.3 | 76.5 | 0.96 (0.95 − 0.98) |
|  |  |  |  |  |
|  | Vernodalol | 11.8 | 10.2 | 0.86 (0.78 − 0.94) |
|  |  |  |  |  |
| 2 | DMSO control | 113.3 | 107.7 | 0.95 (0.93 − 0.97) |
|  |  |  |  |  |
|  | Vernolide | 102.7 | 96.7 | 0.94 (0.93 − 0.96) |
|  |  |  |  |  |
|  | Vernodalol | 23.5 | 19.5 | 0.83 (0.78 − 0.90)* |
|  |  |  |  |  |
| 3 | DMSO control | 116.5 | 112.8 | 0.97 (0.96 − 0.98) |
|  |  |  |  |  |
|  | Vernodalol | 26.8 | 25.7 | 0.96 (0.92 − 0.99) |
|  |  |  |  |  |
| 4 | DMSO control | 80.3 | 76.5 | 0.95 (0.92 − 0.98) |
|  |  |  |  |  |
|  | Vernodalol | 22.3 | 20.8 | 0.91(0.86 − 0.96) |
|  |  |  |  |  |

ESS= early sporogonic stage; DMSO =dimethyl sulfoxide

*The selective impact of vernodalol on the maturation process of ookinete was not reproducible as it is demonstrated by experiment 1, 2, and 3..
