# Supplementary material for: Plasmodium transmission blocking activities of Vernonia amygdalina extracts and isolated compounds
Source: Malar J. 2015 Jul 25;14:288. doi: 10.1186/s12936-015-0812-2 (PMC4513948; doi:10.1186/s12936-015-0812-2)
Supplement: Additional file 2: Table S2. — Ratio of ookinete to total early sporogonic stage counts in microplate wells treated with Vernonia amygdalina fractions. [file 12936_2015_812_MOESM2_ESM.docx]

**Table S2: Ratio of ookinete to total early sporogonic stage counts in microplate wells treated with *Vernonia amygdalina* fractions.**

| **Experiment** | **Test agent** | **Average ESS** | **Average**  **ookinete** | **Ookinete/ESS (%95 CI)** |
| --- | --- | --- | --- | --- |
| 1 | DMSO control | 134.67 | 128 | 0.95 (0.94 −0.96) |
|  |  |  |  |  |
|  | Fr 11 | 0.00 | 0.00 | NA |
|  |  |  |  |  |
|  | Fr 12 | 0.33 | 0.33 | 1.00 |
|  |  |  |  |  |
|  | Fr 13 | 0.67 | 0.67 | 1.00 |
|  |  |  |  |  |
|  | Fr 14 | 0.00 | 0.00 | NA |
|  |  |  |  |  |
| 2 | DMSO control | 219.00 | 212.80 | 0.97 (0.96 −0.98) |
|  |  |  |  |  |
|  | Fr 2 | 221 | 214.67 | 0.97 (0.96 −0.98) |
|  |  |  |  |  |
|  | Fr 3 | 186.33 | 181.33 | 0.97 (0.97 −0.98) |
|  |  |  |  |  |
|  | Fr 5 | 215.00 | 207.67 | 0.97 (0.97 −0.98) |
|  |  |  |  |  |
|  | Fr 7 | 224.33 | 212.00 | 0.95 (0.93 −0.96) |
|  |  |  |  |  |
|  | Fr 9 | 194.67 | 184 | 0.95 (0.94 −0.95) |
|  |  |  |  |  |
|  | Fr 10 | 186.67 | 177.67 | 0.95 (0.94 −0.96) |
|  |  |  |  |  |
|  | Fr 11 | 0.67 | 0.33 | 0.50 |
|  |  |  |  |  |
|  | Fr 12 | 0.67 | 0.33 | 0.50 |
|  |  |  |  |  |
|  | Fr 13 | 0.33 | 0.33 | 1.00 |
|  |  |  |  |  |
|  | Fr 14 | 0.00 | 0.00 | NA |
|  |  |  |  |  |

DMSO =dimethyl sulfoxide; Fr= fraction; NA= not applicable; ESS= early sporogonic stage

NB: fractions 11 to 14 showed almost a complete inhibition of ESS development. In the second experiment, fractions 11 and 12 showed a lower ratio of ookinete to ESS than other test wells however these data do not confirm the stage specificity of the fractions as the change in only one zygote count affects the ratio significantly.
